# International flow of Zambian nurses

**DOI:** 10.1186/1478-4491-7-83

**Published:** 2009-11-11

**Authors:** Naomi Hamada, Jill Maben, Barbara McPake, Kara Hanson

**Affiliations:** 1 Independent Researcher, Gifu, Japan; 2 King's College, London, National Nursing Research Unit, Florence Nightingale School of Nursing and Midwifery, London, UK; 3 Institute for International Health and Development, Queen Margaret University, Edinburgh, UK; 4 Health Economics and Financing Programme, London School of Hygiene and Tropical Medicine, London, UK

## Abstract

This commentary paper highlights changing patterns of outward migration of Zambian nurses. The aim is to discuss these pattern changes in the light of policy developments in Zambia and in receiving countries.

Prior to 2000, South Africa was the most important destination for Zambian registered nurses. In 2000, new destination countries, such as the United Kingdom, became available, resulting in a substantial increase in migration from Zambia. This is attributable to the policy of active recruitment by the United Kingdom's National Health Service and Zambia's policy of offering Voluntary Separation Packages: early retirement lump-sum payments promoted by the government, which nurses used towards migration costs.

The dramatic decline in migration to the United Kingdom since 2004 is likely to be due to increased difficulties in obtaining United Kingdom registration and work permits. Despite smaller numbers, enrolled nurses are also leaving Zambia for other destination countries, a significant new development.

This paper stresses the need for nurse managers and policy-makers to pay more attention to these wider nurse migration trends in Zambia, and argues that the focus of any migration strategy should be on how to retain a motivated workforce through improving working conditions and policy initiatives to encourage nurses to stay within the public sector.

## Introduction

Nurses and midwives constitute the largest of the health professional groups in Zambia and other low-income countries. Therefore it is the attrition and movement of such workers, particularly through migration, that can cripple a health system in sub-Saharan African countries.

**Figure 1:**
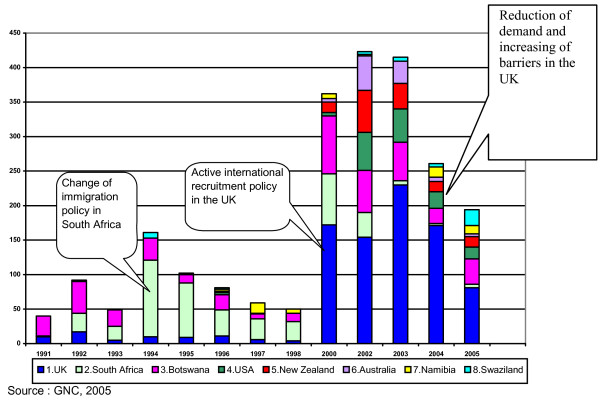
**Number of RNs requesting verifications from the GNC for the top eight destination countries (1991--2005)**.

There has been a considerable volume of analysis and commentary on migration trends of health workers from Africa to the United Kingdom and other well-resourced countries in the early 2000s, but little detailed mapping and analysis with data from African 'sending' countries. Since that period, it is clear that the situation has changed significantly (Fig 1), with dramatic increases in nurse migration from 2000 to 2004.

This paper addresses the factors involved in trends both before and after that change, using a detailed analysis of data from Zambia, 1991-2005. Thus the aim of the paper is to examine the impact of policy on changes in the Zambian nursing workforce, including substantial changes in the migration patterns of Zambian nurses.

## Methods

We used the number of nurses requesting verifications from the General Nursing Council (GNC) in Zambia as an indicator of nurses' intention to leave the country. Primary data at the GNC were hand searched, collated and analysed in Zambia by one of the present authors (NH).

Any nurse wishing to practise abroad must be registered with the professional regulatory authority of the destination country. In the case of the United Kingdom, for example, a verification letter from the GNC in Zambia is required to confirm that the applicant is competent to undertake safe and effective practice when nurses apply for registration with the United Kingdom's Nursing and Midwifery Council (NMC).

Data on Zambian nurses registering with the United Kingdom NMC were also collected and analysed as part of this study. These data were compared with the number of Zambian nurses applying to the Zambian GNC for United Kingdom verification documents.

## Discussion

### Migration trends over time

Figure 1 illustrates the number of Zambian registered nurses (RNs) requesting verification from the GNC for the top eight destination countries (Australia, Botswana, Namibia, New Zealand, South Africa, Swaziland, the United Kingdom of Great Britain and Northern Ireland and the United States of America).

Prior to 2000, South Africa was the most important single destination. Since 2000, the substantial increase in migration is attributable to access to new destination countries such as Australia, New Zealand, the United Kingdom and the United States, in addition to established destination countries such as Botswana and South Africa. Botswana has long been popular, especially among registered midwives (RMs): higher salary, proximity to home, better housing and provision of uniforms were often cited as attracting Zambian nurses [1].

Countries within the Southern African region are also used as stepping stones to migrate to the United Kingdom and elsewhere. By 2000 the United Kingdom replaced South Africa as the most popular destination country.

The dramatic increase from 2000 is also attributable to the active recruitment policy of the United Kingdom's NHS and Zambia's Voluntary Separation Package (VSP), an early retirement lump-sum payment promoted by the government, used by some nurses to pay migration fees and flight costs.

However, since 2004 there has been a dramatic decline in migration, especially to the United Kingdom, the most popular destination country. The overall reduction since 2004 is likely to be related to increased difficulties in obtaining registration and work permits in the United Kingdom.

### Sending country verifications and registration with the United Kingdom NMC

Table 1 compares the number of Zambian nurses applying to the Zambian GNC for United Kingdom verifications with those registering with the NMC in the United Kingdom. The numbers applying for verifications correspond quite closely with the numbers of Zambian nurses registering with the NMC (United Kingdom), with a one-year time lag.

**Table 1:** Comparison of verification numbers in Zambia and registration numbers in the United Kingdom

*Number of nurses applying to GNC (Zambia) for verifications for the United Kingdom*	*4* *(1998)*	*44* *(1999)*	***178*** *(2000)*	***152*** *(2001)*	***167*** *(2002)*	***238*** *(2003)*	***170*** ***(2004)***
Numbers of nurses registering with the NMC (United Kingdom)	15 (1998/1999)	40 (1999/2000)	88 (2000/2001)	**183**(2001/ 2002)	**133** (2002/2003)	**169** (2003/2004)	**162** (2004/2005)

Source: GNC and NMC, 2005. The numbers applying for verifications correspond quite closely with the numbers of Zambian nurses registering with the NMC (United Kingdom), with a one-year time lag.

This time lag is a plausible gap between applying for verification in Zambia and formal admittance to the United Kingdom's NMC register. During this time, nurses must complete paperwork, move to the United Kingdom and undertake three to six months of supervised practice. However, there are some discrepancies between these two datasets, most importantly the 76 nurses who seem not to have registered with the NMC in 2004-2005, despite having applied to the GNC for verification letters in 2003. The shortage of supervised-practice places and greater difficulties in obtaining visas may account for this failure to absorb the supply in 2004-2005 [2], although the will to migrate remained. Some may have registered in 2005-2006, as the registration process is valid for two years. Overall, however, our data suggest that sending country (GNC) verifications are a good indicator of actual migration.

### Ethical recruitment policies

A new South African immigration policy introduced in 1994 may have led fewer nurses to migrate to South Africa between 1994 and 1999. This policy aimed to limit regional recruitment within the Southern African Development Community (SADC) in response to a regional brain drain into South Africa [3] and appears to have affected the choice of destination countries for Zambian nurses.

On the other hand, an active recruitment policy in the United Kingdom seemed to play a substantial role in increasing the migration from 2000. This, however, had a different purpose from the policy in South Africa. In the United Kingdom, the National Health Service (NHS) was keen to fill nurse vacancies and had human resources policies designed to engage in and encourage overseas recruitment, whereas those in South Africa aimed to limit and create an ethical approach to migration.

The United Kingdom did have an ethical recruitment policy, yet interestingly the ethical guidelines issued by the United Kingdom's Department of Health in November 1999 appear to have had no impact: there was a dramatic increase in migration after their implementation. The guidelines specifically state that NHS employers should avoid direct recruitment from South Africa and the Caribbean. This resulted in short-term reductions in recruitment from South Africa and the Caribbean, but recruitment activity may have been displaced to other developing countries, including Zambia [4].

In 2002 the United Kingdom Department of Health released a more detailed Code of Practice; in 2003 it added other countries to a list of less-developed countries to be avoided, including Zambia. The Commonwealth also adopted the Code of Practice for the International Recruitment of Health Workers in 2003. However, there is no direct evidence that these ethical guidelines were effective. Indeed a recent evaluation of the Code of Practice does not identify it as significantly contributing to the recent drop in migration to the United Kingdom [2].

### Enrolled nurses

**Figure 2:**
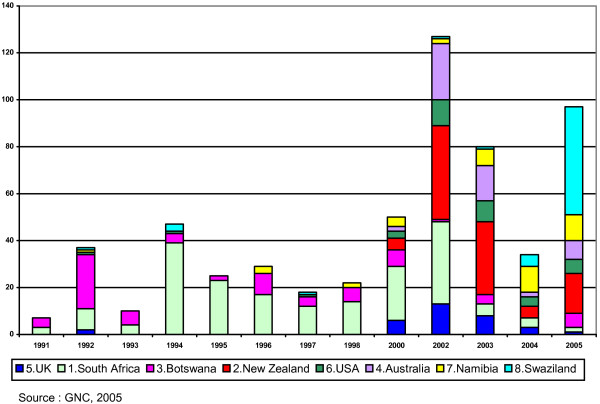
**Number of ENs requesting verifications from the GNC for the top eight destination countries (1991--2005)**.

Although the majority of nurses requesting verifications are RNs, enrolled nurses (ENs) are also leaving Zambia. Figure 2 shows the number of Zambian ENs requesting GNC verifications for the top eight destination countries.

The United Kingdom is the most popular destination country for RNs, but ENs favour New Zealand and, more recently, Swaziland. New Zealand may be more popular among ENs because it retains their cadre, which no longer exists in the United Kingdom, therefore most ENs leave for countries other than the United Kingdom.

In contrast to the reductions in RN verifications in 2004 and 2005, EN verifications increased substantially in 2002 and more recently in 2005. While EN migration mirrored RN migration between 2002 and 2004, by falling sharply, the trends for the two cadres diverged in 2005. It is not clear what the causes of the initial decline were, as the United Kingdom had not been the major recipient country and explanations for RN decline do not therefore apply. It is also not clear why this decline reversed again in 2005 - whether the principal cause was push factors in Zambia or pull factors in the recipient country.

As Fig 1 and Fig 2 show, the majority of nurses requesting verifications are RNs. Discussion of nurse migration has tended to focus on RNs. However, despite the smaller number of enrolled nurses requesting verifications, the analysis of enrolled nurse data presented in this paper is an important new finding and suggests all professional levels of nursing cadres can migrate, not just RNs.

## Conclusion

Comparison between sending country verifications and registration with the United Kingdom NMC provides new insights into nurse migration. Furthermore, our data suggest that the number of migrants is determined by active recruitment policies or those restricting migration in destination countries. South African immigration policy aimed to limit regional recruitment within the Southern African Development Community (SADC) and an active recruitment policy in the United Kingdom seemed to play a substantial role in influencing migration patterns. On the other hand, the ethical guidelines issued by the United Kingdom's Department of Health and the Code of Practice adopted by the Commonwealth appear to have had little or no impact on migration patterns.

For the first time, the data in this paper confirm the declining trend in Zambian nurses migrating to South Africa 1994-1999. This supports Arango's assertion that restrictive entry policies are currently much more influential than differential wages in determining migration [5].

Although migration to South Africa was reduced, the change of immigration policy by one destination country does not significantly affect the number of migrants from a specific country, as long as substantial push factors and migration opportunities in other destination countries remain. For example, migration to the United Kingdom dramatically increased in 2000-2003, even after migration to South Africa was reduced.

In 2006, the United Kingdom removed entry-level staff nurses and senior staff nurses from the shortage-occupation list that allows employers to hire overseas staff more easily [6]. After this rule change, about one third of the United Kingdom members of the Philippines Nurses Association said they were applying for jobs elsewhere, e.g. Australia.

As Figs 1 and 2 suggest, there are many potential destination countries for nurses; agreeing upon a quota of nurses with only the main destination countries will not be sufficient to halt migration trends entirely. Despite the potential effects of restricting migration through multilateral agreements, this kind of control-oriented policy does not address the micro-level issues that are the root causes of migration.

Kingma argues that restrictive immigration policies violate an individual's rights to make international moves, while neglecting the root causes of migration [7]. Policy-makers tend to focus on migration restriction as a retention strategy. However, we suggest the focus of any retention strategies should be on how to retain a motivated workforce through improving working conditions and using policy initiatives to encourage nurses to stay within the public sector [1].

Finally, despite the fact that the majority of migrants are RNs, policy-makers should also pay attention to ENs, who migrate to different destination countries, yet whose migration also contributes to the loss of skilled health workers from low-income countries.

## Competing interests

The authors declare that they have no competing interests.

## Authors' contributions

All authors have been involved in the analysis and writing of this paper. NH was also responsible for the initial data collection at the GNC (Zambia) and NMC and coordinated the writing of this paper. JM and BM were supervisors of the research and KH was an advisory group member. All authors have seen and approved the final version.

## References

[B1] Toyoshi-Hamada N Zambian public sector nurses' incentives and motivation in the context of migration: how to retain Zambian nurses? PhD thesis 2007 London School of Hygiene and Tropical Medicine

[B2] Buchan J The Impact of the Department of Health, England, Code of Practice on International Recruitment 2007 Edinburgh: Queen Margaret University

[B3] Padarath A Chamberlain C McCoy D Ntuli A Rowson M Loewenson R Health Personnel in Southern Africa: Confronting maldistribution and brain drain Equinet Discussion Paper, no.4. Harare: Equinet 2003

[B4] Buchan J International recruitment of nurses: United Kingdom case study 2002 Edinburgh: Queen Margaret University

[B5] Arango J Explaining migration: a critical view International Social Science Journal 2000 52 165 283 296 10.1111/1468-2451.00259

[B6] O'Dowd A Have We Failed Overseas Nurses? Nursing Times 2006 102 43 20 21 17089819

[B7] Kingma M Nurses on the Move 2006 New York: ILR Press Cornell University Press

